# Predicting a Tendency to Use Drugs From Child and Adult Attention Deficit Hyperactivity Disorder Symptoms in Adults

**DOI:** 10.5812/ijhrba.6630

**Published:** 2012-11-26

**Authors:** Mansour Bayrami, Jaber Alizadeh Goradel, Touraj Hashemi, Majid Mahmood-Alilu

**Affiliations:** 1 Department of Psychology, Faculty of Education and Psychology, University of Tabriz, Tabriz, IR Iran

**Keywords:** Adulthood Attention Deficit Hyperactivity Disorder, Substance Abuse, Alcoholism

## Abstract

**Background:**

Adult attention deficit hyperactivity disorder (ADHD) increases the risk of several psychiatric disorders, like substance use disorders (SUDs).

**Objectives:**

This study aimed to predict the tendency for drug use from child and adult ADHD symptoms in adults among male students from Tabriz University, Iran.

**Patients and Methods:**

For this purpose, 361 students were selected via a stratified random sampling from different faculties of Tabriz University. The students completed the Conners Adult ADHD Rating Scale self-report form and subscale (CAARS) questionnaire, Addiction Acknowledgment Scale (AAS) and MacAndrew Alcoholism Revised-Scale (MAC-R).

**Results:**

To analyze the data Pearson correlation and multiple regressions (step by step) were used. Results indicated that there is a significant relationship between scores on the AAS and MAC-R via child and adult ADHD symptoms (P = 0.01). Moreover, we found that those with the highest addiction acknowledgment (13%) exhibited adult ADHD (total) and child ADHD. Alcohol potential (15%) was related to scores of child ADHD and impulsivity.

**Conclusions:**

According to this result behavioral disorders, especially ADHD, have an effect on the tendency to use drugs and therefore the primary treatment of behavioral disorders could prevent future drug abuse.

## 1. Background

Attention deficit hyperactivity disorder (ADHD) is a disorder that has become better recognized in adults during the past few years, with a reported prevalence of 1.0 – 2.5% in the Netherlands (1) and 4.4% in the United States (2). Adults with ADHD suffer from attention and concentration disabilities, hyperactivity or (internal) restlessness, and impulsive behavior. Many people with ADHD also suffer from rapidly changing moods and irritability, resulting in academic and occupational underachievement and recurrent failures. Moreover, comorbidity is common among adults with ADHD. Approximately 75% of adults with this condition may manifest other disorders (3). These may include learning disabilities, anxiety disorders (20 – 30%) or other mood disorders (20 – 30%), personality disorders (25%), and substance use disorders (SUDs) (15 – 45%) (4), facial tics, autism, and behavioral disorders also often coexist with ADHD and persist into adulthood (5). Substance use is particularly troublesome during this period as it often manifests in serious social, legal, academic, behavioral, and family-related problems (6). Adolescents with substance use disorders (SUDs) are also more likely to have co-occurring psychiatric diagnoses compared with adolescents without SUDs. In 2000, substance dependence was estimated to account for $67 billion in economic loss due to crime, social problems, foster care, and other health services (7). For SUD more broadly (ie, combining abuse and dependence), the lifetime prevalence in the same age range varied from 15.3% to 18.0% (2). In fact, there is a sizable body of research suggesting that ADHD is associated with elevated substance use and related disorders (8, 9). In a large (n = 240) case control study, children with ADHD were two times more likely to develop substance dependence disorders than matched controls (10). ADHD was also robustly related (odds ratio N9) to the likelihood of having a SUD in a study of 968 male adolescents in Brazil (11). Thompson et al. assessed 171 adolescents with conduct-disorder (CD) in a residential treatment program and found that ADHD was significantly associated with severe CD and substance problems (12). Similarly, ADHD was associated with severe substance dependence in a sample of 367 clinic-referred male and female adolescents (13). However, null associations between ADHD and substance problems have also been reported. In a sample of 1302, 11 - 15 years old adolescents, ADHD was unrelated to substance use and related problems (14). Similarly, in a prospective study of adolescents diagnosed with ADHD when they were 7 – 11 years old, maltreatment, but not childhood ADHD, independently predicted substance problems (15). There are several reasons that ADHD and substance problems may be related. First, dopamine (DA) neurotransmission is central to current models of ADHD and SUD (16, 17), and methylphenidate (MPH) is a highly efficacious treatment for the core symptoms of ADHD, although recent evidence suggests that the therapeutic response may be time-limited (18). Positron emission tomography (PET) suggests that MPH enhances extracellular DA in the basal ganglia and anterior cingulate gyrus (19). MPH, by virtue of activating positive attention networks and distilling task-irrelevant stimuli, improves attention, vigilance, and motivation (20). Second, a recent review of neuroimaging studies of humans with ADHD and SUD found replicated evidence of blunted striatal DA release and disrupted neural circuitry between the anterior cingulate cortex and striatum with the prefrontal cortex (21). Rodent and non-human primate models suggest the centrality of deficits in response inhibition, including dysfunctional circuitry in the ventro lateral frontal, cingulate cortices, and basal ganglia regions, in both ADHD and SUD (22). Third, the offspring of adults with SUD are more likely to develop psychopathology, including ADHD (23). Elevated substance use problems have also been frequently reported in parents of children with ADHD (24). Finally, the prevalence of psychopathology, including SUD, is higher in first degree relatives of ADHD inclined than in healthy controls (25). Therefore, ADHD and SUD may share common etiological influences, including similar genetic factors (26). This study was conducted in order to investigate the association between adult ADHD (and factors) and child ADHD via the Addiction Acknowledgment and Alcohol Potential Scales. The research questions were as follows:

1) Is there any relationship between adult ADHD (inattention, hyperactivity, impulsivity subtypes, problems with self-concept) and child ADHD via addiction acknowledgment and alcohol potential?

2) Which of the studied variables can better predict addiction acknowledgment and alcohol potential?

## 2. Objectives

The aim of the present study was to predict tendency to use drugs (accepting drugs l and alcohol potential) by child and adult ADHD symptoms among adult students of Tabriz University.

## 3. Patients and Methods

The method of this research is descriptive-correlational. Multivariate regression was used for the statistical analyses. The statistical population of this research was 5129 students in faculties of Tabriz University, the sample was selected from this population. The sample for the present study consisted of 361 male students from the Tabriz University who were selected by stratified random sampling.

### 3.1. Research Instruments

Four questionnaires were used in this study.

1) Conners Adult ADHD Rating Scale self-report form and subscale (CAARS).To date, the Conners Adult ADHD Rating Scale (CAARS) is frequently used in the assessment of adult ADHD (27). The CAARS is available in both a self- and observer-rating form. The short form consists of only 26 items which are rated on a Likert scale from 0 (not at all) to 3 (severe). A validation study tested internal consistency, test-retest reliability, concurrent validity, criterion validity, and the diagnostic utility of the CAARS in a clinical sample of 167 adults (97 males and 70 females). All subjects were referred to an outpatient ADHD clinic for diagnostic assessment. Their mean age was 34.3 years (SD = 11.6). All psychometric quality criteria reached highly satisfactory values (28). Arabgol et al. (29), obtained the Cronbach’s alpha for this scale (0.81). In the current study, the reliability coefficient of the inventory with a sample size of 361 subjects was 0.85.

2) Wender Utah Rating scale (WURS): The Wender-Utah Rating Scale (WURS) was developed to question childhood symptoms of ADHD retrospectively as an aid for the diagnosis of ADHD in adults (30). It is based upon Utah criteria which were developed to diagnose ADHD in adults. First a 61 item five-point Likert type self-assessment scale questioning ADHD symptoms in childhood was developed. The purpose of the WURS is to quantify retrospective self-reports of childhood hyperactive, inattentive, and distractive symptoms, The original study correctly identified 86% of the patients with ADHD, 99% of the normal subjects, and 81% of the depressed patients (30). Internal consistency and test-retest reliability after one month of WURS were reported to be high in university students (31). Sarami-Froushani (32) obtained the coefficients of reliability of the scale (0.95). In the current study, the reliability coefficient of the inventory with a sample size of 361 subjects was 0.90.

3) Mac Andrew Alcoholism-Revised Scale: This measure is a 49-item scale developed with the original MMPI to distinguish alcoholic psychiatric patients from nonalcoholic psychiatric patients. A high MAC-R Scale score is associated with substance abuse potential and other addictive problems such as pathological gambling. At-score cut off of 60 on the MAC-R Scale is suggestive of high addiction potential. The scale seems to be effective with both men and women, as well as inpatients and outpatients (33). Khodayarifard et al. (34) obtained the coefficients of the scale’s reliability about 0.87. In the current study, the reliability coefficient of the inventory with a sample size of 361 subjects was 0.65.

4) Addiction Acknowledgment Scale (AAS): The development of the AAS began with a rational search through the MMPI-2 item pool for items with content indicating substance-abuse problems. Fourteen such items were found. Items not contributing to internal consistency were dropped and replaced by two items that improved scale internal consistency. The AAS is made up of 13 items. Research has shown that the AAS discriminates well between substance abuse samples and samples of either psychiatric patients or normal (35). In this study Khodayarifard et al. (34) obtained the coefficients of reliability of the scale about 0.62. In the current study, the reliability coefficient of the inventory with a sample size of 361 subjects was 0.65.

## 4. Results

Results are presented according to the research questions posed above.

1) Is there any relationship between adult ADHD and factors such as; inattention, hyperactivity, impulsivity, problems with self-concept, and child ADHD via addiction acknowledgment and alcohol potential? In order to answer this question, the Pearson correlation and analysis of regression methods were used. The results demonstrated that the correlation between addiction acknowledgment and alcohol potential via all the predictor’s variables is significant (Table 1).

**Table 1:** Descriptive Statistics and Correlations, (n = 360)

	Mean ± SD	Addiction Acknowledgment	Alcohol Potential
**Alcohol potential**	15.02** ± **3.36		****
**Addiction acknowledgment**	1.99** ± **1.64		
**Adult ADHD**	27.95** ± **10.11	0.341 ^a^	0.283 ^a^
**Inattention**	5.04** ± **2.84	0.300 ^a^	0.174 ^a^
**Hyperactivity**	6.64** ± **2.79	0.239 ^a^	0.211 ^a^
**Impulsivity**	5.008** ± **2.28	0.239 ^a^	0.313 ^a^
**Problems with self-concept**	4.27** ± **2.67	0.323 ^a^	0.192 ^a^
**Child ADHD**	61.67** ± **24.29	0.320 ^a^	0.368 ^a^

Abbreviation: ADHD, attention deficit hyperactivity disorder

^a^P < 0.01

2) Which of the studied variables can better predict addiction acknowledgment and alcohol potential? The second research question was examined by regression analysis, and the results are presented in Table 2 and Table 3. These show that adult ADHD (total) were the strongest predictors and entered the model first, followed by child ADHD. The first predictor accounted for 11% of the variance in addiction acknowledgment (F = 47.113, df = 1359, P ≤ 0.001). The combination of the two predictors accounted for 13% of variance in addiction acknowledgment (F = 9.189, df = 1358, P ≤ 0.001). In Table 3, which shows that child ADHD was the strongest predictor and entered the model first, followed by impulsivity. The first predictor accounted for 13% of the variance in alcohol potential (F = 56.0803, df = 1359, P ≤ 0.001). The combination of the two predictors accounted for 15% of variance in alcohol potential (F = 9.996, df = 1358, P ≤ 0.001).

**Table 2:** Summary of the Regression Model for the Prediction of Addiction Acknowledgment a

	R ^a^	Adjusted R2	R Square Change	F Change	df1	df2	*P* value
**Model 1**	0.341 ^b^	0.114	0.116	47.113	1	359	0.000
**Model 2**	0.372^c^	0.133	0.022	9.189	1	358	0.003

^a^Dependent Variable: Addiction Acknowledgment

^b^Predictors: (Constant), adult ADHD (total)

^c^Predictors: (Constant), adult ADHD (total), child ADHD

**Table 3:** Summary of the Regression Model for the Prediction of Alcohol Potential a

	R ^a^	Adjusted R2	R Square Change	F Change	df1	df2	*P* value
**Model 1**	0.368 ^b^	0.133	0.135	56.080	1	359	0.000
**Model 2**	0.398 ^c^	0.154	0.023	9.996	1	358	0.002

^a^Dependent Variable: alcohol potential

^b^Predictors: (Constant), child ADHD

^c^Predictors: (Constant), child ADHD, impulsivity

## 5. Discussion

To sum up the findings of the current study, addiction acknowledgment was positively predicted by adult ADHD (total) and child ADHD, and alcohol potential was positively predicted by child ADHD and impulsivity. These findings are supported by another study’s results which were obtained from different age groups (8-11). In explaining these findings we can say; child ADHD is a reliable predictor of negative outcomes across academic, social, neuropsychological, and affective domains. Hence, multi finality, where multiple negative outcomes share a common developmental origin, area defining features of ADHD (36). However, far less is known about the prospective contribution of childhood ADHD to subsequent substance use and related disorders (abuse/dependence) than these other domains. To quantitatively characterize the association of ADHD on SUD and to strengthen a potential causal inference by establishing temporal ordering, we focused on prospective longitudinal studies (37). Our meta-analysis provides persuasive evidence of three key findings, (a) childhood ADHD conferred a significant increase in the odds of ever having used nicotine or illicit drugs, but not for alcohol, (b) childhood ADHD prospectively predicted the likelihood of developing adolescent/adult nicotine, alcohol, marijuana, and cocaine use disorders (ie, abuse or dependence), as well as unspecified drug abuse/dependence, and (c) empirical tests of potential moderators for outcomes with heterogeneity in effect size estimates, consisting of demographic or methodological features that varied across studies, were not significant. That is, the reported effect sizes for ADHD and substance problems did not differ significantly by, average age at follow-up, gender, race, sample source (clinic-referred vs school-population-based), or the DSM version used to determine ADHD. In addition to the statistical significance of the association between ADHD and substance problems, we emphasize the size of the effects; children with ADHD were at least 1.5 times more likely to develop SUD across diverse forms of substances, including nearly three times higher for nicotine dependence. Considered together, our results suggest that early ADHD strongly predicts future substance abuse/dependence in adolescence/adulthood and that this association is largely impervious to demographic and methodological factors that varied across each study (38). Consistent with prior research (39), we found high comorbidity rates of adult ADHD symptoms with a broad spectrum of psychiatric disorders, including alcohol abuse/dependence, nicotine dependence, mood disorders, anxiety disorders, somatoform disorders, and pathological gambling. The association between adult ADHD symptoms and substance use disorders may reflect impulsivity, deviant peer groups, comorbid conduct or antisocial personality disorder, and self-medication of individuals with ADHD symptoms, in addition to shared genetic risks among subjects with both disorders (40). In terms of clinical implications, the present findings suggest that the treatment of adult ADHD should involve detailed patient assessments and possible concomitant treatment for highly comorbid conditions such as; alcohol abuse/dependence, nicotine dependence, mood, anxiety and somatoform disorders, and sleep disturbances. It is uncertain whether early successful treatment of childhood ADHD reduces the subsequent risk of psychiatric disorders in adulthood, or whether treatment of adult ADHD affects the severity and course of comorbid disorders, although there is evidence that successful treatment of childhood ADHD reduces the risk of substance abuse (11) as well as childhood symptoms of comorbid disorders. Further studies are required to assess whether the treatment of childhood and adulthood ADHD affects the risk of comorbid conditions during adulthood.
